# Ethanolamine utilization in *Vibrio alginolyticus*

**DOI:** 10.1186/1745-6150-7-45

**Published:** 2012-12-12

**Authors:** Neelam Khatri, Indu Khatri, Srikrishna Subramanian, Saumya Raychaudhuri

**Affiliations:** 1 CSIR-Institute of Microbial Technology, Sector 39A, Chandigarh, India

**Keywords:** Pathogenesis, Ethanolamine, Vibrio, Eut operon, Metabolosome

## Abstract

**Abstract:**

Ethanolamine is used as an energy source by phylogenetically diverse bacteria including pathogens, by the concerted action of proteins from the *eut*-operon. Previous studies have revealed the presence of *eutBC* genes encoding ethanolamine-ammonia lyase, a key enzyme that breaks ethanolamine into acetaldehyde and ammonia, in about 100 bacterial genomes including members of gamma-proteobacteria. However, ethanolamine utilization has not been reported for any member of the *Vibrio* genus. Our comparative genomics study reveals the presence of genes that are involved in ethanolamine utilization in several *Vibrio* species. Using *Vibrio alginolyticus* as a model system we demonstrate that ethanolamine is better utilized as a nitrogen source than as a carbon source.

**Reviewers:**

This article was reviewed by Dr. Lakshminarayan Iyer and Dr. Vivek Anantharaman (nominated by Dr. L Aravind).

## Findings

The relative effectiveness of a microbe as a pathogen depends largely on its ability to survive in different hosts. To achieve this, pathogens employ a multitude of strategies to usurp host-derived nutrients [1]. One such small molecule ethanolamine, present abundantly in host diet, as well as in bacterial and epithelial cells of the mammalian intestine, has been shown to play a contributory role in the pathogenesis of *Salmonella enterica* serotype Typhimurium by acting as a rich source of carbon and nitrogen in the mammalian gut environment [2]. Other than *Salmonella enterica*, a variety of phylogenetically diverse gut and other environmental bacteria can use ethanolamine as a source of carbon, nitrogen and energy [2,3].

**Figure 1:**
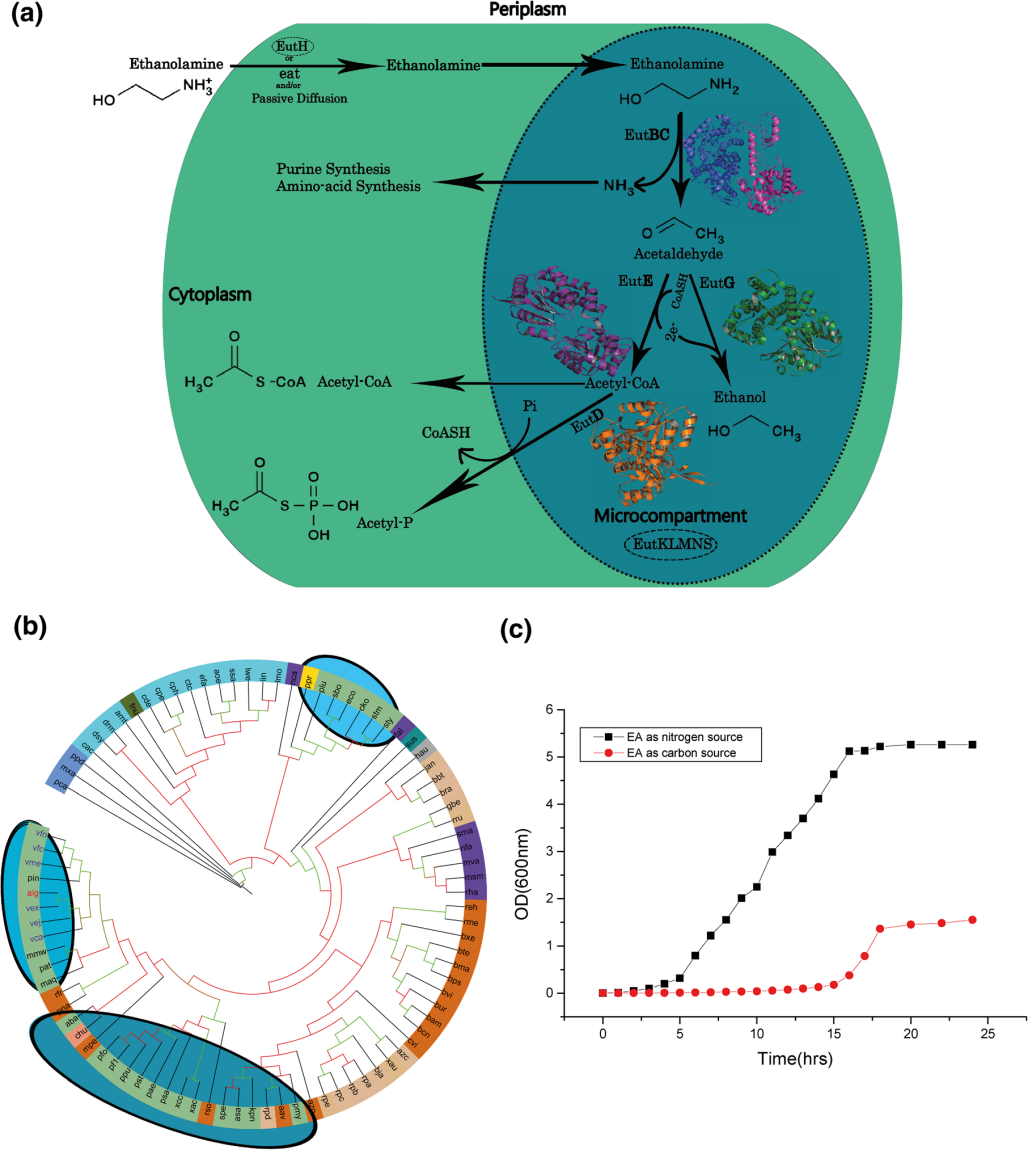
**Ethanolamine utilization in Vibrio spp. a) ***Schematic diagram of ethanolamine utilization:* Ethanolamine is transported into the cytoplasm by the action of transporter proteins EutH, eat and via passive diffusion. Ethanolamine is then broken down into ammonia and acetaldehyde by the EutBC complex. Ammonia is further utilized by the purine and amino-acid synthesis pathways. Acetaldehyde is converted into Acetyl-P and Acetyl-CoA by the action of EutD, EutE and EutG proteins. The microcompartment made up of the EutKLMNS structural proteins help sequester volatile metabolites like acetaldehyde and the enzymes required for ethanolamine utilization. The microcompartment and EutH proteins which are not present in *V. alginolyticus* are circled with dotted lines. Structural diagrams of homology models of the various enzymes are shown at their respective locations in the pathway. The EutB protein adopts a TIM barrel fold; the EutC protein adopts an anticodon-binding domain like fold while EutD, EutE and EutG adopt the Rossmann fold. **b) ***Maximum likelihood evolutionary tree based on the EutB sequences:* The branch color represents bootstrap support: green >80% and red <50%. The colored circle marks bacterial clades: burly wood, *Alphaproteobacteria*; chocolate, *Betaproteobacteria*; dark sea green, *Gammaproteobacteria*; corn flower blue, *Deltaproteobacteria*; cyan, *Firmicutes*; blue violet, *Actinobacter*; dark cyan, *Acidobacter*; dark olive green, *Fusobacter*; grey, *Chlorophlexi*; and dark salmon, *Bacteroidetes*. The ovals with shaded background mark the clades that contain *Gammaproteobacteria*. Abbreviations of organism names are as per the Additional file 2: Figure S1. **c) ***Growth curve of Vibrio alginolyticus in minimal media containing ethanolamine:* 25 ml minimal media was inoculated with a 1:100 dilution of an exponentially grown culture of *Vibrio alginolyticus* strain V105. The culture was grown at 30°C and growth at various time points was monitored spectrophotometrically by measuring OD_600nm_.

In recent past, a great deal of information on the process of utilization of ethanolamine has been obtained by studying *Salmonella enterica* as a model organism. These studies suggest that the concerted action of 17 proteins help convert ethanolamine into more metabolically suitable molecules [4]. Further, it has also been seen in *Salmonella enterica* that all essential proteins for ethanolamine metabolism are clustered into a multiprotein complex known as the metabolosome, which is reminiscent of the bacterial microcompartment [5]. After its entry into the cytoplasm by the action of the transporter proteins EutH and/or eat [2,4] and possibly by passive diffusion [6], ethanolamine is broken down into ammonia and acetaldehyde by ethanolamine ammonia lyase encoded by the genes *eutB* and *eutC*. This process requires the cofactor adenosylcobalamin, which is produced from cobalamin by the corrinoid cobalamin adenosyltransferase protein encoded by *eutT*[7]. While ammonia serves as a cellular source of reduced nitrogen, the acetaldehyde is further converted to acetyl-CoA, by an aldehyde oxidoreductase encoded by *eutE,* and enters the carbon pool of the cell. Acetyl-CoA can also be modified into acetylphosphate by a phosphotransacetylase EutD. Alternatively, acetaldehyde can be converted to alcohol by another oxidoreductase encoded by *eutG* [Figure 1a. Apart from these enzymes, other proteins contribute indirectly in the ethanolamine utilization process. For example, *eutA* encodes a reactivating factor for ethanolamine ammonia lyase EutBC in *Salmonella*[8] and EutJ acts as a chaperone for EutG and EutE [9,10]. The *eut* operon is positively regulated by EutR, a DNA binding protein of the AraC family of transcription regulators. Additionally, *eut* operons of the *Firmicutes and Enterobacteriaceae* encode few other proteins such as EutP and EutQ whose functions are not clear.

The genus *Vibrio* of the class *Gammaproteobacteria* is an ecologically and metabolically diverse group autochthonous to the marine, estuarine and freshwater environment [11]. These bacteria are involved in nutrient cycling, degrade hydrocarbons and maintain a commensal-to-pathogenic relationship with many diverse animals including vertebrates and invertebrates. Though sequences of genes encoding EutB and EutC have been reported in nearly 100 bacterial genomes (including several *Gammaproteobacteria* such as *Escherichia coli*, *Acinetobacter baumannii*, *Pseudoalteromonas atlantica*) [4], no functional *eut* operon has been reported in *Vibrio*. This prompted us to search for the genes from the *eut* operon in the available *Vibrio* genomes.

Sequence analysis with PSI-BLAST (default parameters) using as query the EutB and EutC proteins of *Salmonella enterica* revealed similar proteins in *Photobacterium profundum* 3TCK and in several species of *Vibrio* such as *Vibrio alginolyticus* 12G01, *Vibrio sp.* Ex25, *Vibrio furnissi* CIP 102972, *Vibrio furnissi* NCTC 11218, *Vibrio sp.* EJY3, *Vibrio metschnikovii* CIP 69.14 and *Vibrio caribbenthicus* ATCC BAA-2122. Additional searches using as query the sequences of other *Salmonella enterica eut*-operon proteins within these *Photobacterium and Vibrio* species were carried out. *Photobacterium profundum* 3TCK, *Vibrio alginolyticus* 12G01, *Vibrio furnissi* CIP 102972, *Vibrio sp.* EJY3 and *Vibrio metschnikovii* CIP 69.14 contained the maximum number of *eut*-operon-related proteins viz., EutABCDEGHJKLMNPQRST, EutBCDEGPQR, EutBCDEGHKR, EutBCDEGHJKLMNS and EutBCDEGJPQR, respectively [See Additional file 1: Table S1]. A STRING database [12] analysis of the *Vibrio sp.* Ex25 genome revealed the presence of an *eat* gene that codes for an ethanolamine permease. PSI-BLAST searches using this sequence as query confirmed the presence of the *eat* gene in *Photobacterium profundum* 3TCK, *Vibrio alginolyticus* 12G01, *Vibrio furnissi* CIP 102972, *Vibrio furnissi* NCTC 11218, *Vibrio sp.* EJY3, and *Vibrio caribbenthicus* ATCC BAA-2122. *Vibrio metschnikovii* CIP 69.14 interestingly lacks both the EutH and the eat proteins. *Photobacterium profundum* 3TCK, *Vibrio sp.* Ex25, *Vibrio furnissi* CIP 102972 and *Vibrio furnissi* NCTC 11218 have both EutH and eat proteins suggesting the presence of both the long and short versions of the *eut* operon as reported in other organisms previously [2,13]. As *Vibrio alginolyticus* was readily available with us, we used it as a model system for experimental verification of our prediction that some *Vibrio spp.* are likely to metabolize ethanolamine.

*Vibrio alignolyticus* has gained attention in the recent years as a prominent fish pathogen. It is a halophilic and mesophilic rod-shaped flagellated Gram-negative bacterium and causes high mortality vibriosis in various fish species such as sea bream, grouper, large yellow croaker, kuruma prawn [14]. It exhibits fairly low pathogenicity for humans and there are only a few clinical cases where it was found associated with superficial wounds, ear or eye infections [13]. In recent years, a consorted effort has been made to understand the biology, and in particular the virulence mechanism of this bacterium. Taxonomically, the organism is closely related to *Vibrio harveyi* and *Vibrio parahemolyticus* and shared many cellular features including virulence determinants which are required to establish infection in fish cells [15-18].

In order to understand the evolutionary relationship of the *Vibrio eut* proteins vis-a-vis other *proteobacteria,* we generated a maximum likelihood tree based on the EutB and EutC proteins of *Vibrio alignolyticus* and other representative *proteobacteria* [Figure 1b; Additional file 2: Figure S1]. The protein sequences aligned using PCMA [19], were used to construct Bayesian [20] and ML [21] trees using Topali [22] and edited on the iTOL server [23]. The EutB- and EutC-based trees were largely in conformity with each other and with those reported earlier [4]. *Alphaproteobacteria*, *Betaproteobacteria*, *Deltaproteobacteria* and *Gammaproteobacteria* subdivisions of *proteobacteria* form distinct clustered clades that are not matted within. Interestingly, in both the EutB and EutC trees, *Vibrio alginolyticus* (*alg*) and other *Vibrio spp.* considered in this study are closest to the extremophilic sea-ice bacteria *Psychromonas ingrahamii 37* (*pin*) [Figure 1b*.* The clade distinctly contains most of the *Gammaproteobacteria* with the exception of *Photorhabdus luminescens subsp. salmonicida* A449, *Citrobacter koseri* ATCC BAA-895, *Escherichia coli str.* K12 *substr.* MG1655, *Salmonella enterica subsp. enterica serovar Typhi str.* CT18, *Salmonella typhimurium* LT2, *Shigella boydii* Sb227, *Photobacterium profundum* 3TCK that form a separate clade. *Pseudomonas aeruginosa PAO1, Xanthomonas axonopodis pv. citri str. 306, Xanthomonas campestris pv. campestris str. 8004, Pseudomonas fluorescens Pf-5, Pseudomonas fluorescens PfO-1, Pseudomonas stutzeri A1501, Pseudomonas putida KT2440, Pseudomonas syringae pv. tomato str. DC3000* form a third clade. The clades also contains some B*etaproteobacteria* such as *Methylibium petroleiphilum* PM1, *Rhodoferax ferrireducens* T118, *Polaromonas naphthalenivorans* CJ2, *Acidovorax avenae subsp. citrulli* AAC00-1 and *Ralstonia solanacearum* GMI1000 which cluster together with the *Gammaproteobacteria* revealing the close similarity of the EutB and EutC proteins from these two classes of *proteobacteria.*

The multiple sequence alignment obtained using PCMA [19] of all EutB and EutC proteins revealed that the residues essential for cobalamin binding and EutBC activity are conserved in *Vibrio alginolyticus.* We also analyzed the genomic positions of the *eut*-operon genes. Unlike *Salmonella enterica*, where all the *eut* genes are part of a single operon with the exception of *eutR,* in *Vibrio alginolyticus*, only *eutB* and *eutC* are next to each other. We could however identify several other *eut* genes in *Vibrio alginolyticus.* In order to understand the evolutionary model of the distribution of the *eut* genes, we performed a comprehensive survey of the *eut* operon proteins in all known genomes (unique species) of the *Photobacterium and Vibrio* families. A total of 32 genomes were selected, and based on 16S rRNA similarity were grouped into clades as per the classification proposed by Sawabe *et.al.*[17]. The genomes represent 9 of the 14 clades described previously. Sequence analysis with PSI-BLAST (default parameters) using as query the various *eut* operon proteins of *Salmonella enterica*, as mentioned previously, helped retrieve the corresponding proteins from the respective members of the *Photobacterium and Vibrio* families. Our data strongly supports a scenario where the entire *eut* operon was already present in the ancestor of the *Photobacterium* and *Vibrio* families followed by differential loss of several genes in most of the currently sequenced members of the *Photobacterium and Vibrio* families. Indeed, the sole exception is the genome of *Photobacterium profundum* 3TCK, where the whole set of 17 genes of the *eut* operon present in *Salmonella enterica* along with the *eat* permease gene are completely conserved. In other genomes, we observe the presence of several, but not all, of the *eut* operon proteins and the conserved proteins share significant sequence similarity. It is interesting to note that the carbon utilizing enzymes (EutD, EutE and EutG) are particularly well conserved among all the *Vibrionaceae* and *Photobacterium* families and share a high sequence identity among the different *Vibrionaceae* and *Photobacterium* members [Additional file 1: Table S1]. It appears that there has been extensive deletion of the *eutBC* genes as well as those coding for the metabolosome structural components (*eutKLMNS*) in a large majority of the members of the *Vibrionaceae* and *Photobacterium* families.

In order to ascertain whether *Vibrio alginolyticus* can metabolize ethanolamine, we performed a growth experiment in minimal media supplemented with ethanolamine as energy source using a previously described method [6]. Briefly, overnight Luria broth grown culture of *Vibrio alginolyticus* was diluted and grown to an A_600nm_ of 0.6 at 30°C in Luria broth. The culture was again diluted 100-fold in M9 minimal salt medium containing KH_2_PO_4_ (15 g l^-1^), Na_2_PO_4_.7H_2_O (64 g l^-1^), NaCl (2.5 g l^-1^) supplemented with 0.1 mM CaCl_2_, 2 mM MgSO_4,_ 200 nM vitamin B12 (cyanocobalamin). To evaluate the ability of *Vibrio algoinolyticus* to utilize ethanolamine as a nitrogen source, 82 mM of ethanolamine hydrochloride was added along with 0.4% glucose to minimal medium. To test the ability of the bacterium to use ethanolamine as a carbon source, 82 mM of ethanolamine hydrochloride and NH_4_Cl (5.0 g l^-1^) were added to minimal medium. Cultures were then incubated with agitation (200 rpm) at 30°C and growth was monitored over 24 h. Our data suggests that *Vibrio alginolyticus* can utilize ethanolamine as a source of nitrogen and to a lesser extent as a carbon source [Figure 1c. As ethanolamine can be used as a source of nitrogen by *Vibrio alginolyticus*, we wanted to compare the growth of the bacterium in presence of an alternate nitrogen source. Growth of *Vibrio alginolyticus* was monitored in minimal media containing NH_4_Cl as a nitrogen source. Our data suggests that growth of *Vibrio alginolyticus* in ethanolamine is slightly better than in NH_4_Cl [Additional file 3: Figure S2].

Previously, it has been shown that enterohaemorrhagic *Escherichia coli* (EHEC) can utilize ethanolamine solely as a nitrogen source, and ethanolamine ammonia lyase alone is sufficient to support growth of the bacterium in minimal media containing ethanolamine [6]. Mutant strains of EHEC deleted for *eutD, eutE*, *eutG* exhibit similar growth phenotype to those of the wild type strain, indicating the functional dispensability of these genes in the utilization of ethanolamine as a nitrogen source. Although, the *eutD, eutE* and *eutG* genes that play a role in utilization of ethanolamine as a carbon source are present in the genome of *Vibrio alginolyticus*, we notice that ethanolamine is not utilized as a carbon source as effectively as it is used as a source of nitrogen. This difference can be rationalized by the absence of the *eutMNLK* genes in *Vibrio alginolyticus* which code for the metabolosome. The lack of a functional metabolosome would prevent the sequestering of both the metabolite acetaldehyde as well as the enzymes required for utilizing carbon as an energy source. Our findings are in accord with a previously reported study where it was shown that *eutMNLK* mutant strain of *Salmonella enterica* lacking a functional metabolosome is unable to grow on ethanolamine as the sole source of carbon and energy [24]. It should be highlighted that *Vibrio sp.* EJY3 contains both transporter proteins EutH and eat in addition to all components of a functional metabolosome (EutMNLK) and enzymes required for nitrogen and carbon utilization from ethanolamine. It would be of interest to examine the ability of this organism to utilize ethanolamine both as a carbon and a nitrogen source.

To summarize, ethanolamine ammonia lyase is sufficient for the utilization of ethanolamine as a nitrogen source while a functional metabolosome together with all other relevant enzymes are necessary to efficiently utilize ethanolamine as a carbon source. Our work is the first report describing the presence of a functional, albeit minimal, ethanolamine utilization operon in *Vibrio* species. By considering *Vibrio alginolyticus* as a model organism, we have evaluated the capacity of this bacterium to utilize ethanolamine and our study highlights a new dimension of the metabolic potential of *Vibrio spp.*

## Abbreviations

Eut: Ethanolamine utilization; eat: Ethanolamine permease; EA: Ethanolamine; EHEC: Enterohaemorrhagic *Escherichia coli*; PCMA: Profile Consistency Multiple sequence Alignment; FFAS: Fold and Function Assignment System.

## Competing interests

The authors declare that they have no competing interests.

## Authors’ contributions

SRC conceived the idea, NK carried out growth experiments, IK and SKS performed bioinformatics analysis, SRC and SKS wrote the manuscript. All the authors read and approved the manuscript.

## Reviewers’ names

This article was reviewed by Dr. Lakshminarayan Iyer (Reviewer 1) and Dr. Vivek Anantharaman (Reviewer 2, nominated by Dr. L Aravind).

Reviewers’ comments

Reviewer’s report

Title: Ethanolamine utilization in *Vibrio alginolyticus*

Version: 1 Date: 29 October 2012

## Reviewer number: 1

## Report form

Khatri and colleagues report the presence of ethanolamine catabolism genes in several *Vibrio* species, and additionally study ethanolamine usage by *Vibrio alginolyticus* in minimal media. The discovery of these genes in *Vibrio* species, *per se*, isn’t surprising and has been reported by various *Vibrio* genome annotation projects. However, in light of the correlation between ethanolamine usage and gut pathogenesis, and the potential role for ethanolamine as a signaling molecule, the study might interest *Vibrio* specialists. The sequence and phylogenetic analysis are straight-forward and easily reproducible, and the growth kinetics are self-explanatory. A few comments follow.

1) What is the evolutionary model of the distribution of eut genes in *Vibrio*? An ancestral presence followed by differential loss, or multiple independent lateral transfers? The authors might address this by including eut genes from all representatives of the *Vibrionaceae*-family (including those from Photobacterium) in the phylogenetic analysis.

2) The growth kinetics only show that ethanolamine is used by *V. alginolyticus* as a sole nitrogen or carbon source. There is no support for Vibrio preferentially using ethanolamine as a nitrogen source. Preferential usage implies choice between multiple substrates, which the experiments do not test.

3) Minor comment: On page 4, the authors use the term lower- and higher-life-forms. It is unclear what is lower or higher in the list of animals that follow. It might be more precisely reworded as "diverse animals including vertebrates and invertebrates".

Quality of written English: Acceptable.

## Authors’ response

We thank Dr. Lakshminarayan Iyer for his insightful comments.

1) In order to understand the evolutionary model of the distribution of *eut* genes in *Vibrio*, we searched for the presence of all *eut* operon proteins in available genomes of the *Vibrionaceae* and *Photobacterium* families. We observe the presence of several of the *eut* operon proteins in all genomes [Additional file 1: Table S1]. Our analysis strongly supports the presence of an ancestral *eut* operon followed by differential loss of several genes in both the *Vibrionaceae* and *Photobacterium* families. It is interesting to note that the carbon utilizing enzymes (EutD, EutE and EutG) are particularly well conserved among all the *Vibrionaceae* and *Photobacterium* families and share a significant sequence identity. It appears that there has been extensive deletion of the *eutBC* genes as well as those coding for the metabolosome structural components (*eutKLMNS*) in a large majority of the members of the *Vibrionaceae* and *Photobacterium* families. Interestingly in the *Photobacterium* family, *Photobacterium profundum* 3TCK contains all the proteins (EutABCDEGHJKLMNPQRST and eat) of the *eut* operon while other members lack several of the *eut* operon proteins including EutBC.

2) We agree with the reviewer about the usage of the term “preferential” and have avoided using it throughout the manuscript. In order to evaluate the choice between multiple nitrogen sources, we have performed independent growth analysis in minimal media containing either ammonium chloride or ethanolamine. We observe that *Vibrio alginolyticus* grows slightly better in a medium containing ethanolamine as nitrogen source as compared to the medium containing ammonium chloride.

3) Regarding the minor comment on page 4, we have modified the text as suggested by the reviewer.

## Reviewer number 2

## Report form

The authors have presented a study of Ethanolamine utilization in *Vibrio*. I have a few comments:

Identifying the *vibrio* eut operon proteins through sequence analysis is trivial. For example, a PSI-Blast search seeded with EutB of *Salmonella enterica* recovers EutB of *Vibrio alginolyticus* in the first iteration with an e-value of 7e-151. Moreover, PFAM profiles easily identify the domain in the protein. Hence, the sentence “there is no report of the existence of these genes in Vibrio genomes” is superfluous. This should be toned down to something akin to “no previous studies on the vibrio eut genes”.

The authors have performed ethanolamine utilization experiment on *Vibrio alginolyticus*. To make the paper more complete and tie-in the eut operon discussion to the experiments, the authors should consider doing the ethanolamine utilization experiment on eut gene deletion mutants in Vibrio. Such an experiment would provide direct evidence of the involvement of the eut genes in ethanolamine utilization in Vibrio.

Figure 1b, can be improved by showing the biochemical pathway with the chemical structures and reactions drawn out.

Quality of written English: Acceptable

## Authors’ response

We thank Dr. Vivek Anantharaman for his insightful comments. While we agree with him that the identification of the *eut* operon genes in *Vibrio* is a trivial task, we would like to highlight that no previously published work has identified a functional *eut* operon in any *Vibrio* or *Photobacterium* genome as positive for ethanolamine utilization. This is possibly because a large majority of the *Vibrio* genomes available today do not contain genes that code for EutBC and several other *eut* operon proteins that are thought to be essential for ethanolamine utilization. Our primary interest in this study was to establish the capacity of some *Vibrios* to utilize ethanolamine as an energy source.

We agree with both the reviewers that the sentence “…. there is no report of the existence of these genes in *Vibrio* genomes” is incorrect and so we are modifying the sentence to “…. no functional *eut* operon has been reported in *Vibrio*”.

As pointed out by both the reviewers, there is a large degree of similarity between *eut* genes of *Vibrio* and *Salmonella*. As *Salmonella eut* operon and corresponding mutants have been studied extensively, we believe similar results will be obtained for *Vibrio*. However, this part will be addressed separately. In this brief Discovery Note, we would like to highlight that the genome of *Vibrio alginolyticus* (and some other *Vibrios*) contain genes of the *eut* operon and the organism is capable of harvesting energy from ethanolamine as a substrate.

Minor change

Figure 1b has been improved with all the chemical structures drawn out as suggested.

## Supplementary Material

Additional file 1 **Table S1. ***eut operon proteins present in Photobacterium and Vibrio genomes***:** A comprehensive list of all *eut* operon proteins from the available genomes of *Photobacterium* and *Vibrio spp*. The first column has the species names of *Photobacterium* and *Vibrio spp.* grouped into various clades. The first row contains the names of the proteins of the *eut* operon viz., EutA: Reactivating Factor, EutB: Ethanolamine ammonia lyase large subunit, EutC: Ethanolamine ammonia lyase small subunit, EutD: Phosphotransacetylase, EutE: Aldehyde oxidoreductase, EutG: Alcohol dehydrogenase, EutH: Transport protein, EutJ: Putative chaperonin, EutK: Metabolosome structural protein, EutL: Metabolosome structural protein, EutM: Metabolosome structural protein, EutN: Metabolosome structural protein, EutP: Ethanolamine utilization protein, EutQ: Ethanolamine utilization protein, EutR: Transcriptional regulator, EutS: Metabolosome structural protein, EutT: Corrinoid cobalamin adenosyltransferase and eat: Ethanolamine permease. PSI-BLAST searches were carried out for all 32 genomes using protein sequences of the *Salmonella entrica eut* operon as queries. The accession numbers correspond to the various eut proteins from these species. The sequence identity between various *eut* operon proteins were obtained using the blast2seq program and are provided in separate sheets. Sequence pairs for which no significant alignment could be obtained are denoted as “N”. The rows and columns list the names of the organisms used in the analysis, which have been grouped into various clades based on 16S rRNA similarity. The clades are colored yellow (*Photobacterium*), dodgerblue (*Splendidus*), cornflowerblue (*Corallilyticus*), blueviolet (*Scopthalmi*), gold (*Anguillarum*), sandybrown (*Vulnificus*), plum (*Harveyi*), yellowgreen (*Orientalis*), palevioletred (*Cholerae*) respectively. *Vibrio nigripulchritudo* and *Vibrio shilonii*, which could not be assigned to any known clades are colored pink. Click here for file

Additional file 2 **Figure S1. ***Maximum likelihood evolutionary tree based on the EutC sequences*: The branch color represents bootstrap support: green >80% and red <50%. The colored circle marks bacterial clades: burly wood, *Alphaproteobacteria*; chocolate, *Betaproteobacteria*; dark sea green, *Gammaproteobacteria*; corn flower blue, *Deltaproteobacteria*; cyan, *Firmicutes*; blue violet, *Actinobacter*; dark cyan, *Acidobacter*; dark olive green, *Fusobacter*; grey, *Chlorophlexi*; and dark salmon, *Bacteroidetes*. The ovals with shaded background mark the clades that contain *Gammaproteobacteria*. The taxa names represented in tree are abbreviated forms of: **Alphaproteobacteria**- **bja**: *Bradyrhizobium japonicum* USDA 110, **bbt**: *Bradyrhizobium sp.* BTAi1, **bra**: *Bradyrhizobium sp.* ORS278, **rpe**: *Rhodopseudomonas palustris* BisA53, **rpd**: *Rhodopseudomonas palustris* BisB5, **rpc**: *Rhodopseudomonas palustris* BisB18, **rpa**: *Rhodopseudomonas palustris* CGA009, **rpb**: *Rhodopseudomonas palustris* HaA2, **xau**: *Xanthobacter autotrophicus* Py2, **azc**: *Azorhizobium caulinodans* ORS 571, **jan**: *Jannaschia sp.* CCS1, **gbe**: *Granulibacter bethesdensis* CGDNIH1, **rru**: *Rhodospirillum rubrum* ATCC 11170; **Betaproteobacteria**- **azo**: *Azoarcus sp*. BH72, **reh**: *Ralstonia eutropha* H16, **rme**: *Ralstonia metallidurans* CH34, **rso**: *Ralstonia solanacearum* GMI1000, **aav**: *Acidovorax avenae subsp. citrulli* AAC00-1, **bcn**: *Burkholderia cenocepacia* AU 1054, **bam**: *Burkholderia cepacia* AMMD, **bma**: *Burkholderia mallei* ATCC 23344, **bps**: *Burkholderia pseudomallei* K96243, **bur**: *Burkholderia sp.* 383, **bte**: *Burkholderia thailandensis* E264, **bvi**: *Burkholderia vietnamiensis* G4, **bxe**: *Burkholderia xenovorans* LB400, **cvi**: *Chromobacterium violaceum* ATCC 12472, **mpe**: *Methylibium petroleiphilum* PM1, **rfr**: *Rhodoferax ferrireducens* T118, **pna**: *Polaromonas naphthalenivorans* CJ2; **Gammaproteobacteria**- **asa**: *Aeromonas salmonicida subsp. salmonicida* A449, **plu**: *Photorhabdus luminescens subsp. laumondii* TTO1, **spe**: *Serratia proteamaculans* 568, **cko**: *Citrobacter koseri* ATCC BAA-895, **eco**: *Escherichia coli* str. K-12 substr. MG1655, **maq**: *Marinobacter aquaeolei* VT8, sty: *Salmonella enterica subsp. enterica serovar Typhi* str. CT18, **stm**: *Salmonella typhimurium* LT2, **sbo**: *Shigella boydii* Sb227, **aba**: *Acinetobacter baumannii* ATCC 17978, mmw: *Marinomonas sp*. MWYL1, **xac**: *Xanthomonas axonopodis pv. citri* str. 306, **xcc**: *Xanthomonas campestris pv. campestris* str. 8004, **kpn**: *Klebsiella pneumoniae subsp. pneumoniae* MGH 78578, **pat**: *Pseudoalteromonas atlantica* T6c, **pmy**: *Pseudomonas mendocina* ymp, **pae**: *Pseudomonas aeruginosa* PAO1, **pfl**: *Pseudomonas fluorescens* Pf-5, **pfo**: *Pseudomonas fluorescens* PfO-1, **psa**: *Pseudomonas stutzeri* A1501, **ppu**: *Pseudomonas putida* KT2440, **pst**: *Pseudomonas syringae* pv. tomato str. DC3000, **pin**: *Psychromonas ingrahamii* 37, **alg**: *Vibrio alginolyticus*, **ppr: ***Photobacterium profundum* 3TCK, **vex**: *Vibrio sp.* Ex25, **vfn**: *Vibrio furnissii* NCTC 11218, **vfc**: *Vibrio furnissii* CIP 102972, **vej**: *Vibrio sp.* EJY3, **vca**: *Vibrio caribbenthicus* ATCC BAA-2122, **vme**: *Vibrio metschnikovii* CIP 69.14*; ***Deltaproteobacteria**- **mxa**: *Myxococcus xanthus* DK 1622, **pca**: *Pelobacter carbinolicus* DSM 2380, **ppd**: *Pelobacter propionicus* DSM 2379; **Firmicutes**- **lwe**: *Listeria welshimeri* serovar 6b str. SLCC5334, **lin**: *Listeria innocua* Clip11262, **lmo**: *Listeria monocytogenes* EGD-e, **amt**: *Alkaliphilus metalliredigens* QYMF, **aoe**: *Alkaliphilus oremlandii* OhILAs, **cde**: *Clostridium difficile* 630, **cpe**: *Clostridium perfringens* ATCC 13124, **cph**: *Clostridium phytofermentans* ISDg, **ctc**: *Clostridium tetani* E88, **cac**: *Clostridium acetobutylicum* ATCC 824, **dsy**: *Desulfitobacterium hafniense* Y51, **drm**: *Desulfotomaculum reducens* MI-1, **efa**: *Enterococcus faecalis* V583, **ssa**: *Streptococcus sanguinis* SK36; **Actinobacteria**- **fal**: *Frankia alni* ACN14a, **sma**: *Streptomyces* avermitilis MA-4680, **msm**: *Mycobacterium smegmatis* str. MC2 155, **mva**: *Mycobacterium vanbaalenii* PYR-1, **nfa**: *Nocardia farcinica* IFM 10152, **rha**: *Rhodococcus sp*. RHA1, **nca**: *Nocardioides sp*. JS614; **Acidobacteria**- **sus**: *Solibacter usitatus* Ellin6076; **Bacteriodetes**- **chu**: *Cytophaga hutchinsonii* ATCC 33406; **Fusobacteria**- **fnu**: *Fusobacterium nucleatum* subsp. *nucleatum* ATCC 25586; **Chloroflexi**- **hau**: *Herpetosiphon aurantiacus* ATCC 23779 Click here for file

Additional file 3 **Figure S2.** Growth curve of *V. alginolyticus* in minimal media containing NH_4_Cl as a nitrogen source: 25 ml minimal media was inoculated with a 1:100 dilution of an exponentially grown culture of *V.alginolyticus* strain V105. The culture was grown at 30°C and growth at various time points was monitored spectrophotometrically by measuring OD_600nm_. Click here for file

## References

[B1] Rohmer L Hocquet D Miller SI Are pathogenic bacteria just looking for food? Metabolism and microbial pathogenesis Trends Microbiol 2011 19 341 348 10.1016/j.tim.2011.04.003 21600774 PMC3130110

[B2] Garsin DA Ethanolamine utilization in bacterial pathogens: roles and regulation Nat Rev Microbiol 2010 8 290 295 10.1038/nrmicro2334 20234377 PMC2950637

[B3] Blackwell CM Scarlett FA Turner JM Ethanolamine catabolism by bacteria, including Escherichia coli Biochemical Society transactions 1976 4 495 497 793895 10.1042/bst0040495

[B4] Tsoy O Ravcheev D Mushegian A Comparative genomics of ethanolamine utilization Journal of bacteriology 2009 191 7157 7164 10.1128/JB.00838-09 19783625 PMC2786565

[B5] Eric Kofoid CR Igor S John R The 17 gene ethanolamine (eut) operon of Salmonella typhimurium encodes five homologues of carboxysome shell proteins Journal of bacteriology 1999 181 10.1128/jb.181.17.5317-5329.1999 PMC94038 10464203

[B6] Bertin Y Girardeau JP Chaucheyras-Durand F Lyan B Pujos-Guillot E Harel J Martin C Enterohaemorrhagic Escherichia coli gains a competitive advantage by using ethanolamine as a nitrogen source in the bovine intestinal content Environ Microbiol 2011 13 365 377 10.1111/j.1462-2920.2010.02334.x 20849446

[B7] Sheppard DE Penrod JT Bobik T Kofoid E Roth JR Evidence that a B12-adenosyl transferase is encoded within the ethanolamine operon of Salmonella enterica Journal of bacteriology 2004 186 7635 7644 10.1128/JB.186.22.7635-7644.2004 15516577 PMC524904

[B8] Mori K Bando R Hieda N Toraya T Identification of a reactivating factor for adenosylcobalamin-dependent ethanolamine ammonia lyase Journal of bacteriology 2004 186 6845 6854 10.1128/JB.186.20.6845-6854.2004 15466038 PMC522198

[B9] Igor S Andreas J Baumler J Hefron F Ethanolamine Utilization in Salmonella typhimurium: Nucleotide Sequence, Protein Expression, and Mutational Analysis of the cchA cchB eutE eutJ eutG eutH Gene Cluster Journal of bacteriology 1995 177 1357 1366 7868611 10.1128/jb.177.5.1357-1366.1995 PMC176743

[B10] Penrod JT Mace CC Roth JR A pH-sensitive function and phenotype: evidence that EutH facilitates diffusion of uncharged ethanolamine in Salmonella enterica Journal of bacteriology 2004 186 6885 6890 10.1128/JB.186.20.6885-6890.2004 15466042 PMC522209

[B11] Haley BJ Grim CJ Hasan NA Choi SY Chun J Brettin TS Bruce DC Challacombe JF Detter JC Han CS Comparative genomic analysis reveals evidence of two novel Vibrio species closely related to V. cholerae BMC Microbiol 2010 10 154 10.1186/1471-2180-10-154 20507608 PMC2889950

[B12] Szklarczyk D Franceschini A Kuhn M Simonovic M Roth A Minguez P Doerks T Stark M Muller J Bork P The STRING database in 2011: functional interaction networks of proteins, globally integrated and scored Nucleic acids research 2011 39 D561 D568 10.1093/nar/gkq973 21045058 PMC3013807

[B13] Janda JMPC Bryant RG Abbott SL Current perspectives on the epidemiology and pathogenesis of clinically significant Vibrio spp Clin Microbiol Rev 1988 1 245 267 3058295 10.1128/cmr.1.3.245 PMC358049

[B14] Liu H Gu D Cao X Liu Q Wang Q Zhang Y Characterization of a new quorum sensing regulator luxT and its roles in the extracellular protease production, motility, and virulence in fish pathogen Vibrio alginolyticus Arch Microbiol 2012 194 439 452 10.1007/s00203-011-0774-x 22130678

[B15] Cao X Wang Q Liu Q Liu H He H Zhang Y Vibrio alginolyticus MviN is a LuxO-regulated Protein and Affects Cytotoxicity Towards Epithelioma Papulosum Cyprini (EPC) Cells J Microbiol Biotechnol 2010 20 271 280 20208429

[B16] Cao X Wang Q Liu Q Rui H Liu H Zhang Y Identification of a luxO-regulated extracellular protein Pep and its roles in motility in Vibrio alginolyticus Microb Pathog 2011 50 123 131 10.1016/j.micpath.2010.12.003 21167274

[B17] Sawabe T Kita-Tsukamoto K Thompson FL Inferring the evolutionary history of vibrios by means of multilocus sequence analysis Journal of bacteriology 2007 189 7932 7936 10.1128/JB.00693-07 17704223 PMC2168739

[B18] Zhao Z Chen C Hu CQ Ren CH Zhao JJ Zhang LP Jiang X Luo P Wang QB The type III secretion system of Vibrio alginolyticus induces rapid apoptosis, cell rounding and osmotic lysis of fish cells Microbiology 2010 156 2864 2872 10.1099/mic.0.040626-0 20576689

[B19] Pei J Sadreyev R Grishin NV PCMA: fast and accurate multiple sequence alignment based on profile consistency Bioinformatics 2003 19 427 428 10.1093/bioinformatics/btg008 12584134

[B20] Huelsenbeck JP FR MRBAYES: Bayesian inference of phylogenetic trees. Bioinformatics Bioinformatics 2001 17 754 755 10.1093/bioinformatics/17.8.754 11524383

[B21] Guindon S Gascuel O A Simple, Fast, and Accurate Algorithm to Estimate Large Phylogenies by Maximum Likelihood Systematic Biology 2003 52 696 704 10.1080/10635150390235520 14530136

[B22] Milne I Lindner D Bayer M Husmeier D McGuire G Marshall DF Wright F TOPALi v2: a rich graphical interface for evolutionary analyses of multiple alignments on HPC clusters and multi-core desktops Bioinformatics 2009 25 126 127 10.1093/bioinformatics/btn575 18984599 PMC2638937

[B23] Letunic I Bork P Interactive Tree Of Life v2: online annotation and display of phylogenetic trees made easy Nucleic acids research 2011 39 W475 W478 10.1093/nar/gkr201 21470960 PMC3125724

[B24] Brinsmade SR Paldon T Escalante-Semerena JC Minimal functions and physiological conditions required for growth of Salmonella enterica on ethanolamine in the absence of the metabolosome Journal of bacteriology 2005 187 8039 8046 10.1128/JB.187.23.8039-8046.2005 16291677 PMC1291257

