# Supplementary figures and images for: Ethanolamine utilization in Vibrio alginolyticus
Source: Biol Direct. 2012 Dec 12;7:45. doi: 10.1186/1745-6150-7-45 (PMC3542024; doi:10.1186/1745-6150-7-45)

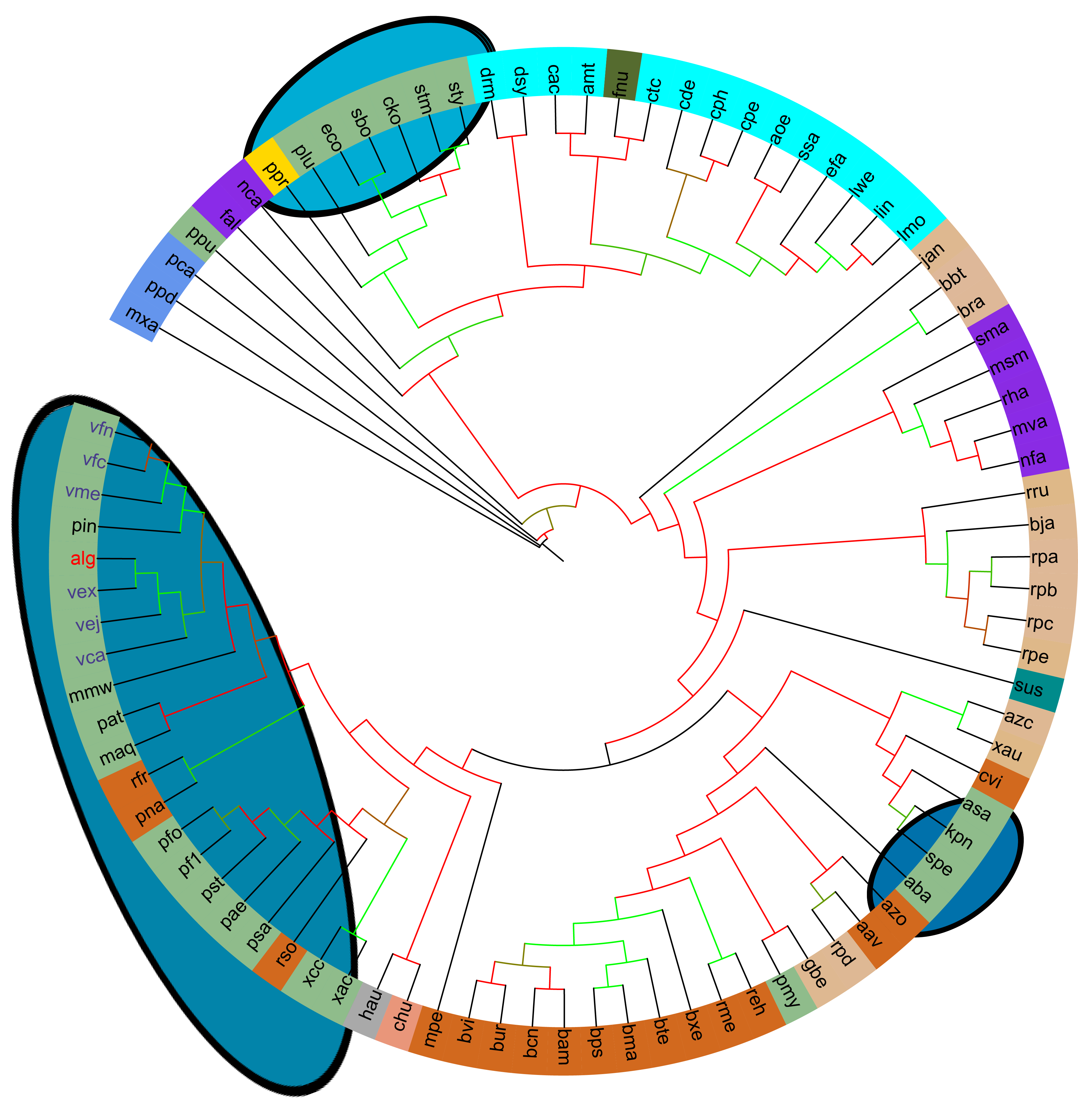

Supplement: Additional file 2 — Figure S1. Maximum likelihood evolutionary tree based on the EutC sequences: The branch color represents bootstrap support: green >80% and red <50%. The colored circle marks bacterial clades: burly wood, Alphaproteobacteria; chocolate, Betaproteobacteria; dark sea green, Gammaproteobacteria; corn flower blue, Deltaproteobacteria; cyan, Firmicutes; blue violet, Actinobacter; dark cyan, Acidobacter; dark olive green, Fusobacter; grey, Chlorophlexi; and dark salmon, Bacteroidetes. The ovals with shaded background mark the clades that contain Gammaproteobacteria. The taxa names represented in tree are abbreviated forms of: Alphaproteobacteria- bja: Bradyrhizobium japonicum USDA 110, bbt: Bradyrhizobium sp. BTAi1, bra: Bradyrhizobium sp. ORS278, rpe: Rhodopseudomonas palustris BisA53, rpd: Rhodopseudomonas palustris BisB5, rpc: Rhodopseudomonas palustris BisB18, rpa: Rhodopseudomonas palustris CGA009, rpb: Rhodopseudomonas palustris HaA2, xau: Xanthobacter autotrophicus Py2, azc: Azorhizobium caulinodans ORS 571, jan: Jannaschia sp. CCS1, gbe: Granulibacter bethesdensis CGDNIH1, rru: Rhodospirillum rubrum ATCC 11170; Betaproteobacteria- azo: Azoarcus sp. BH72, reh: Ralstonia eutropha H16, rme: Ralstonia metallidurans CH34, rso: Ralstonia solanacearum GMI1000, aav: Acidovorax avenae subsp. citrulli AAC00-1, bcn: Burkholderia cenocepacia AU 1054, bam: Burkholderia cepacia AMMD, bma: Burkholderia mallei ATCC 23344, bps: Burkholderia pseudomallei K96243, bur: Burkholderia sp. 383, bte: Burkholderia thailandensis E264, bvi: Burkholderia vietnamiensis G4, bxe: Burkholderia xenovorans LB400, cvi: Chromobacterium violaceum ATCC 12472, mpe: Methylibium petroleiphilum PM1, rfr: Rhodoferax ferrireducens T118, pna: Polaromonas naphthalenivorans CJ2; Gammaproteobacteria- asa: Aeromonas salmonicida subsp. salmonicida A449, plu: Photorhabdus luminescens subsp. laumondii TTO1, spe: Serratia proteamaculans 568, cko: Citrobacter koseri ATCC BAA-895, eco: Escherichia coli str. K-12 substr. [file 1745-6150-7-45-S2.png]

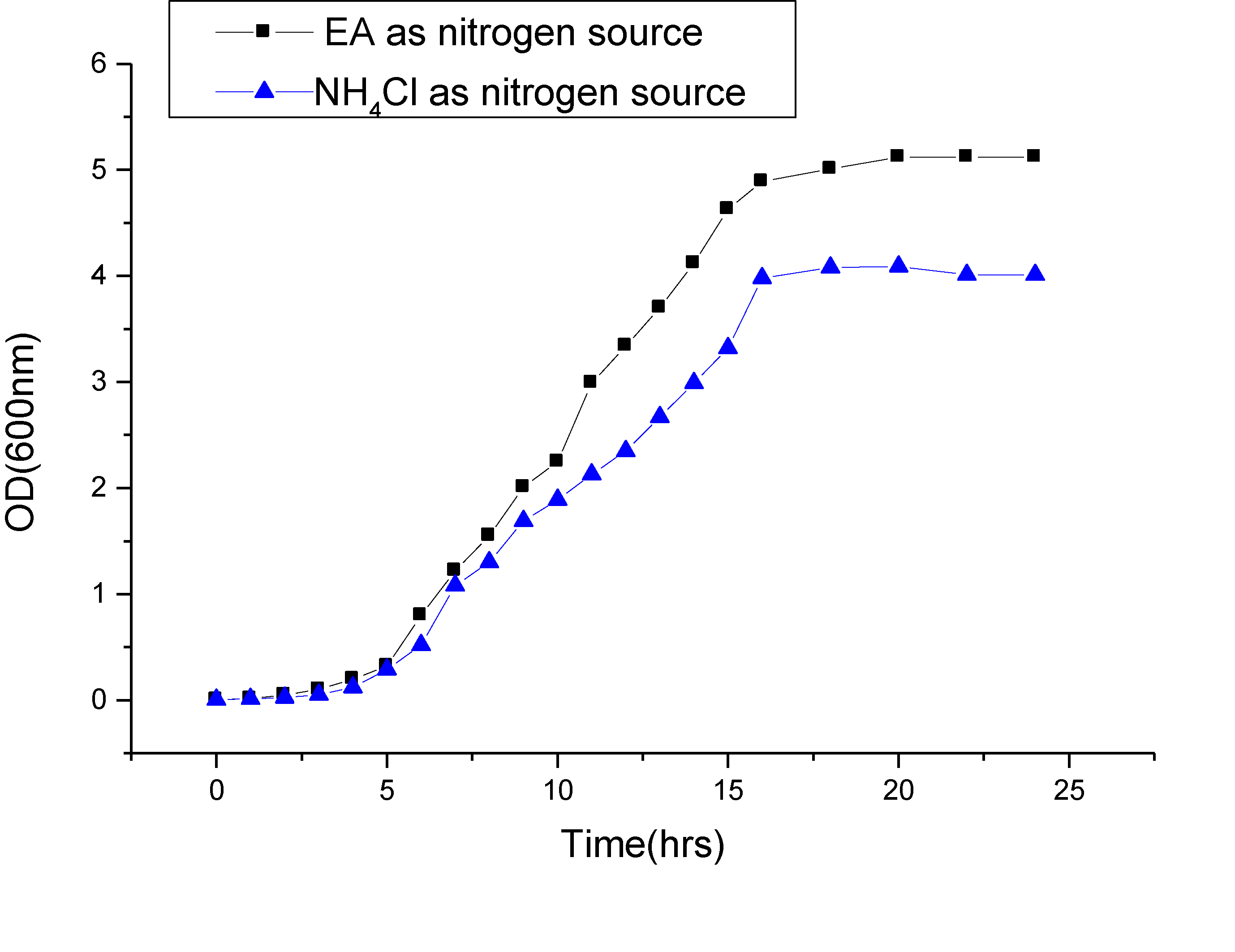

Supplement: Additional file 3 — Figure S2. Growth curve of V. alginolyticus in minimal media containing NH4Cl as a nitrogen source: 25 ml minimal media was inoculated with a 1:100 dilution of an exponentially grown culture of V.alginolyticus strain V105. The culture was grown at 30°C and growth at various time points was monitored spectrophotometrically by measuring OD600nm. [file 1745-6150-7-45-S3.png]
